# Vildagliptin in addition to metformin improves retinal blood flow and erythrocyte deformability in patients with type 2 diabetes mellitus – results from an exploratory study

**DOI:** 10.1186/1475-2840-12-59

**Published:** 2013-04-08

**Authors:** Christine Berndt-Zipfel, Georg Michelson, Markus Dworak, Michael Mitry, Andrea Löffler, Andreas Pfützner, Thomas Forst

**Affiliations:** 1 Institute for Clinical Research and Development, Parcusstrasse 8, Mainz, 55116, Germany; 2 Interdisciplinary Centre for Ophthalmic Preventive Medicine and Imaging, Friedrich-Alexander-University, Erlangen, Germany; 3 Novartis Pharma GmbH, Clinical and Regulatory Affairs, Nürnberg, Germany

**Keywords:** Erythrocyte deformability, Retinal blood flow, Glucose control, Vildagliptin, Glimepiride

## Abstract

Numerous rheological and microvascular alterations characterize the vascular pathology in patients with type 2 diabetes mellitus (T2DM). This study investigated effects of vildagliptin in comparison to glimepiride on retinal microvascular blood flow and erythrocyte deformability in T2DM.

Fourty-four patients with T2DM on metformin monotherapy were included in this randomized, exploratory study over 24 weeks. Patients were randomized to receive either vildagliptin (50 mg twice daily) or glimepiride individually titrated up to 4 mg in addition to ongoing metformin treatment. Retinal microvascular blood flow (RBF) and the arteriolar wall to lumen ratio (WLR) were assessed using a laser doppler scanner. In addition, the erythrocyte elongation index (EI) was measured at different shear stresses using laserdiffractoscopy.

Both treatments improved glycaemic control (p < 0.05 vs. baseline; respectively). While only slight changes in RBF and the WLR could be observed during treatment with glimepiride, vildagliptin significantly increased retinal blood flow and decreased the arterial WLR (p < 0.05 vs. baseline respectively). The EI increased during both treatments over a wide range of applied shear stresses (p < 0.05 vs. baseline). An inverse correlation could be observed between improved glycaemic control (HbA1c) and EI (r = −0.524; p < 0.0001) but not with the changes in retinal microvascular measurements.

Our results suggest that vildagliptin might exert beneficial effects on retinal microvascular blood flow beyond glucose control. In contrast, the improvement in erythrocyte deformability observed in both treatment groups, seems to be a correlate of improved glycaemic control.

## Background

Type 2 diabetes mellitus (T2DM) is associated with numerous vascular and hemorheological abnormalities which merge together in an unproportional high risk for the development of micro- and macrovascular complications like retinopathy, nephropathy, neuropathy, or cardiovascular disease. The assessment of retinal microvascular architecture and the investigation of retinal arterial blood flow allows to detect early vascular abnormalities in patients with T2DM even before the clinical manifestation of diabetic retinopathy [1-3]. In recent studies, a close association could be found between retinal microvascular abnormalities and an increased risk for the development of nephropathy, neuropathy, myocardial infarction, or stroke [4-8].

Recently, dipeptidyl-peptidase IV (DPP-IV) inhibitors have been introduced in the treatment of T2DM. A couple of studies suggested pleiotropic effects beyond metabolic control for this class of drugs. Treatment with DPP-IV inhibitors was found to improve myocardial and endothelial function, to improve blood lipids, to lower blood pressure and to improve markers of renal function [9-16]. In vitro studies demonstrated that DPP-IV is expressed in endothelial cells, and the inhibition of DPP-IV reduced the microvascular tone through direct mediation of the nitric oxide system [17]. Therefore, it seems conceivable that glucose-independent effects of DPP-IV inhibition might be mediated through GLP-1 receptor signalling and /or direct inhibition of the enzyme DPP-IV in vascular, renal, or retinal cells. Based on the different mode of action, these effects might not be applicable to other antidiabetic treatments like K-ATP-channel blocker such as sulfonylureas.

The aim of this exploratory study was to investigate the effect of vildagliptin in comparison to glimepiride as add-on to metformin on retinal microvascular blood flow, retinal microvascular architecture and erythrocyte deformability in type 2 diabetic patients inadequately controlled on metformin monotherapy.

## Methods

This single-centre, randomized, open-label, parallel study compared microvascular and hemorheological effects of treatment with either vildagliptin or glimepiride in type 2 diabetic patients pre-treated with metformin. To be considered eligible, patients had to be aged 30–80 years with an HbA1c in the range of 6.5 to 9.5%. The main exclusion criteria were myocardial infarction or stroke within 6 months prior to study enrolment; impaired hepatic or renal function; moderate or proliferative diabetic retinopathy, more than one unexplained episode of severe hypoglycemia within 6 months; pre-treatment with other anti-diabetic drugs with the exception of metformin within the last 3 months and uncontrolled hypertension (systolic blood pressure >160 and/or diastolic blood pressure >90 mmHg).

The study was performed in compliance with Good Clinical Practice and all applicable national laws and regulations. All patients provided written informed consent and the study was approved by an appropriate independent ethics committee.

Eligible patients were randomized to vildagliptin or glimepiride in a 1:1 ratio. Patients received 50 mg vildagliptin twice daily. Glimepiride was administered in the morning with an individual dose titration in the range of 0.5 – 4 mg to achieve best possible glycaemic control as judged by the investigator. At baseline, after 12 and 24 weeks of treatment, patients entered the study site in the morning after an overnight fast of at least eight hours. Fasting blood samples were obtained for the measurement of blood glucose, HbA1c, adiponectin, and the determination of erythrocyte deformability. In addition, all patients underwent retinal fundoscopy and retinal microvascular assessments.

### Measurement of erythrocyte deformability

Blood cell deformability was measured using a laser-assisted optical rotational cell analyzer by determining the elongation index (EI). Laserdiffractoscopy was performed using the Rheodyn SSD shear stress diffractometer (Myrenne GmbH, Roetgen, Germany). The method of laserdiffractoscopy has been described in detail previously [18]. The applied shear stress was electronically regulated and consists of 8 increasing shear stress ranges (0.3; 0.6; 1.2; 3; 6; 12; 30; 60 Pa). The measurement detects scattered light intensities along orthogonal axes (A, B) of red blood cells within the laser diffraction light cone. The erythrocyte elongation index (EI) was calculated by the following equation: *EI*(*%*) = ((*A* − *B*)/(*A* + *B*)) * 100. To compare the EI over the applied shear stress range between both treatment groups, the area under the curve from 0.3 to 60 Pa (AUC_0.3–60_) was calculated using the trapezoidal method.

### Measurement of retinal microvascular blood flow (RBF) and retinal arteriolar wall to lumen ratio (WLR)

Retinal capillary blood flow was assessed using scanning laser doppler flowmetry at 670 nm (Heidelberg Retina Flowmeter, Heidelberg Engineering, Germany). A retinal sample of 2.56 × 0.64 × 0.30 mm was scanned within 2 seconds at a resolution of 256 points × 64 lines × 128 lines. The confocal technique of the device ensured that only the capillary blood flow of the superficial retinal layer of 300 μm was measured. Measurements were performed in the juxtapapillary area of both eyes, 2 to 3 mm temporally to the optic nerve; the average from 3 singular measurements was taken.

Analysis of perfusion images was performed offline with automatic full-field perfusion imaging analysis. This led to a perfusion map excluding vessels with a diameter of >30 μm, without lines with saccades, and without pixels with inadequate reflectivity. The mean retinal capillary blood flow was calculated in the area of interest and expressed as arbitrary units.

Analysis of vessel diameters was performed offline with automatic full field perfusion imaging analysis (SLDF version 3.7) [19,20]. Outer arteriole diameter (AD) was measured in reflection images, and lumen diameter (LD) was measured in perfusion images. The wall to lumen ratio (WLR) was calculated as (AD-LD)/LD.

The laser scanning records were stored electronically and sent to a central reading centre (Interdisciplinary Centre for Ophthalmic Preventive Medicine and Imaging (IZPI) of the Friedrich-Alexander-University Erlangen-Nürnberg, Germany), for the measurement of retinal microvascular blood flow and the calculation of the retinal wall to lumen ratio (WLR). This reading centre was blinded for all other study procedures.

### Laboratory measurements

Blood glucose levels were determined using an electrochemical biosensor (Hitado, Möhnesee, Germany). Plasma adiponectin was measured using ELISA (total human adiponectin, TECOmedical) and HbA1c was measured by HPLC (Menarini Diagnostics, Neuss, Germany).

### Statistical analysis

This study was designed as an exploratory study aimed to provide new data for thesis generation. No a priory confirmatory sample size estimation has been performed. All study endpoints have been analyzed with equal priority in a non-confirmatory, exploratory sense. RBF and WLR were assessed in a central reading centre in a blinded fashion. All other study endpoints were assessed in an open label approach. All study results were evaluated using primarily descriptive statistics. Inferential statistics was used to compare results from baseline to endpoint within both treatment groups. Differences in means of study endpoints were tested by Student’s t-test. In case of not equal variances in the data, the results of the Welch approximation was taken into account as result of the unpaired comparison. Results are presented as mean ± SD. Significance was set at a p-value less than 0.05. Data processing was performed with the software modules of SPSS (Statistical package for analysis in social sciences, release 19.0, SPSS Inc., Chicago, USA).

## Results

Table 1 summarizes the demographics and baseline characteristics for the 44 patients included in the final analysis. Both study groups were comparable with regard to gender, age, HbA1c, and BMI. Patients in the vildagliptin group were found to have a slightly longer duration of T2DM compared to the patients in the glimepiride group.

**Table 1:** Baseline demographics and baseline characteristics of the study groups

	**Glimepiride**	**Vildagliptin**
n	22	22
Male / female	13 / 9	15 / 7
Age (years)	60 ± 7	57 ± 9
Duration of diabetes (years)	6.1 ± 4.4	8.4 ± 9.0 ^$^
HbA1c (%)	7.3 ± 0.6	7.4 ± 0.7
BMI (kg/m^2^)	33.3 ± 6.7	34.6 ± 5.9

mean ± SD; ^$^ = p < 0.05 in between both groups.

As shown in Table 2, fasting blood glucose and HbA1c levels decreased continuously in both study groups from baseline to the end of the observational period. In patients treated with vildagliptin a reduction in body weight and an increase in adiponectin levels could be observed, while patients treated with glimepiride increased body weight with no change in adiponectin levels.

**Table 2:** Investigational parameters at baseline, after 12 and 24 weeks of treatment in the observational groups

	**Baseline**		**12 weeks**		**24 weeks**	
	**Glimepiride**	**Vildagliptin**	**Glimepiride**	**Vildagliptin**	**Glimepiride**	**Vildagliptin**
HbA1c (%)	7.28 ± 0.59	7.41 ± 0.74	6.70 ± 0.50 *	6.95 ± 0.82 *	6.57 ± 0.45 *	6.74 ± 0.75 *
Fasting BG (mmol/L)	8.2 ± 1.9	8.4 ± 1.5	7.0 ± 1.2 *	7.6 ± 1.9	7.0 ± 1.2 *	7.4 ± 1.4 *
Adiponectin (μg/mL)	5.6 ± 3.1	5.0 ± 3.5	5.4 ± 2.8	5.5 ± 4.1	5.7 ± 2.7	5.7 ± 4.1 *
Body weight (kg)	93.7 ± 19.6	99.3 ± 14.9	94.4 ± 18.9	98.7 ± 15.0	95.5 ± 19.0	97.6 ± 14.3 *
RBF (AU)	74.2 ± 5.3	75.1 ± 5.8	74.7 ± 6.9	75.3 ± 5.8	76.1 ± 7.6	77.9 ± 5.9 *
WLR (AU)	0.47 ± 0.08	0.46 ± 0.06	0.45 ± 0.09	0.43 ± 0.07	0.43 ± 0.08	0.40 ± 0.06 *
EI AUC_0.3–60_ (AU*min)	2536 ± 228	2568 ± 196	2505 ± 256	2570 ± 170	2682 ± 240 *	2623 ± 210 *

BG =blood glucose; RBF =Retinal blood flow; WLR = arteriolar wall to lumen ratio; EI AUC_0.3–60_ = area under curve for the elongation index in the shear stress range of 0.3-60 Pa; mean ± SD; * = p < 0.05 vs. baseline.

**Figure 1:**
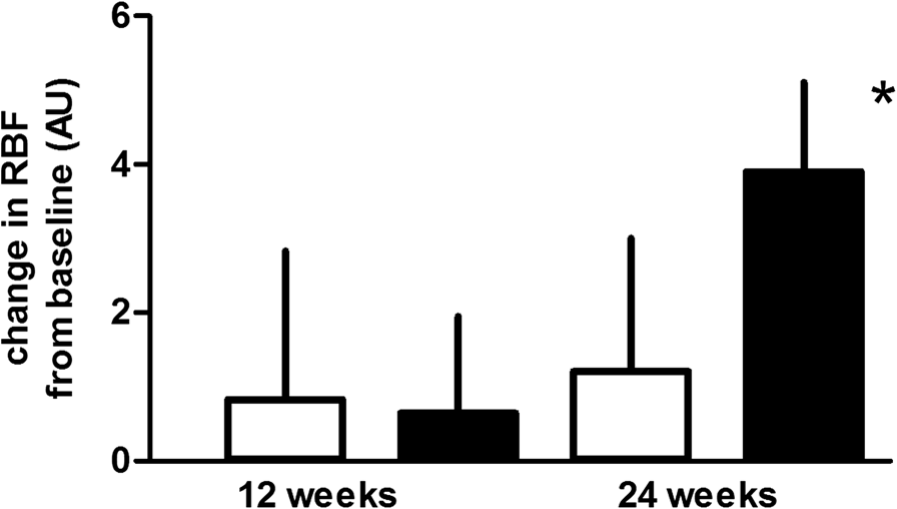
Mean change from baseline in retinal blood flow (RBF) during glimepiride and vildagliptin treatment after 12 and 24 weeks of treatment (□ = glimepiride; ■ = vildagliptin; mean ± SEM; * = p < 0.05 vs. baseline).

**Figure 2:**
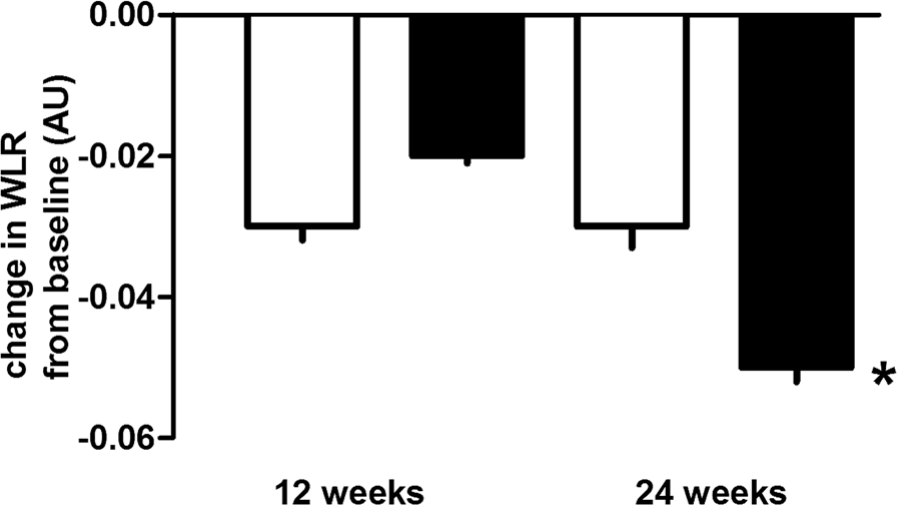
Mean change from baseline in retinal arteriolar wall to lumen ratio (WLR) during glimepiride and vildagliptin treatment after 12 and 24 weeks of treatment (□ = glimepiride; ■ = vildagliptin; mean ± SEM; * = p < 0.05 vs. baseline).

In both groups, an increase in retinal blood flow (RBF) and a decrease in the retinal arteriolar wall to lumen ratio (WLR) could be observed (Figures 1 and 2). After 24 weeks of treatment the increase in RBF and the decrease in the WLR reached statistical significance during treatment with vildagliptin, but not during treatment with glimepiride. No association was found between changes in glucose, HbA1c, or adiponectin levels with the parameters of RBF or arteriolar WLR.

In contrast to the findings in retinal blood flow, the erythrocyte elongation index (EI) improved significantly in both treatment groups over a wide range of applied shear stresses (Table 3). The area under the curve calculated for the shear stress range between 0.3 and 60 Pa increased from 2536 ± 228 to 2682 ± 240 AU*min (p < 0.0001) during treatment with glimepiride and from 2568 ± 196 to 2623 ± 210 AU*min (p < 0.0001) during treatment with vildagliptin. A linear inverse correlation could be observed between fasting blood glucose levels and the EI (r = −0.417; p < 0.0001), and between the HbA1c and the EI (r = −0.524; p < 0.0001). A slight positive correlation was found between adiponectin levels and the EI (r = 0.31; p < 0.001).

**Table 3:** Erythrocyte deformability – elongation index (EI) at different shear stress rates at baseline and after 12 and 24 weeks of treatment in both treatment groups

	**Baseline**		**12 weeks**		**24 weeks**	
**Shear stress (Pa)**	**Glimepiride**	**Vildagliptin**	**Glimepiride**	**Vildagliptin**	**Glimepiride**	**Vildagliptin**
0.3	0.65 ± 0.68	0.86 ± 0.45	0.93 ± 0.80	0.87 ± 0.63	1.65 ± 1.40 *	1.54 ± 0.91 *
0.6	2.66 ± 1.14	2.55 ± 1.24	2.37 ± 1.37	2.65 ± 1.34	3.76 ± 2.13	3.67 ± 1.73 *
1.2	8.53 ± 2.25	8.80 ± 2.00	7.96 ± 2.38	8.79 ± 2.29	10.11 ± 2.66 *	10.24 ± 2.90 *
3	20.89 ± 3.60	21.71 ± 3.24	20.09 ± 3.68	21.53 ± 3.28	24.13 ± 3.74 *	23.95 ± 3.14 *
6	30.50 ± 3.95	31.33 ± 3.45	29.81 ± 4.17	31.15 ± 3.25	33.80 ± 4.13 *	33.20 ± 3.32 *
12	38.62 ± 4.01	39.36 ± 3.34	37.95 ± 4.33	39.28 ± 3.02	41.37 ± 4.12 *	40.59 ± 3.33 *
30	46.43 ± 3.86	47.07 ± 3.38	45.94 ± 4.47	47.14 ± 2.82	48.80 ± 4.13 *	47.71 ± 3.59
60	50.12 ± 3.86	50.75 ± 3.48	49.70 ± 4.36	50.89 ± 2.97	52.69 ± 4.25 *	51.32 ± 4.05

mean ± SD; * = p < 0.05 vs. baseline.

No severe hypoglycemic event was observed during the study. Symptomatic hypoglycemic episodes were reported in 29 cases during treatment with metformin and glimepiride and in 2 cases during treatment with metformin and vildagliptin. Both treatments were overall well-tolerated. No serious adverse event with causal relationship was reported during the study. Infections were recorded in 17.4% of patients during treatment with metformin and glimepiride and in 18.2% during treatment with metformin and vildagliptin. Gastrointestinal disorders were reported in 17.4% of patients during treatment with metformin and glimepiride and in 27.3% during treatment with metformin and vildagliptin.

## Discussion

The burden of T2DM is driven by the development of micro- and macrovascular complications. Vascular dysfunction, changes in the vascular architecture, and hemorheological alterations are early features associated with obesity, metabolic syndrome and the development of T2DM. Remodeling of arterioles and small arteries is an early feature in retinal vascular pathology, and are often found even before the clinical diagnosis of diabetic retinopathy [1,4,21]. The retina offers the unique approach to visualize human microcirculation non-invasively and safely in vivo. Retinal vascular damage was shown to be predictive for the development of micro- and macrovascular complications [5,6,22-24]. A decreased retinal blood flow could be observed in T2DM in association with diabetic nephropathy [25], and an increase in the wall to lumen ratio of retinal arteries was found in association with increased blood pressure, and in patients with a history of cerebrovascular or cardiovascular events [7,8,26]. In a recent study, it was suggested that increased retinal arteriolar WLR may be a reflection of an altered endothelial-mediated release of nitric oxide [27].

Recently, GLP-1 receptor agonists and DPP-IV inhibitors have been introduced in the treatment of T2DM. Treatment with the DPP-IV inhibitor vildagliptin was shown to be well tolerated, to reduce glucose excursions, and to improve the functional capacity of beta cell [28-30]. Apart from the metabolic effects, treatment with DPP-IV inhibitors is supposed to exert several pleiotropic activities which might modulate vascular function in T2DM [31,32]. GLP-1 receptors are widely expressed in pancreatic, kidney, lung, brain, myocardial, and endothelial cells. There is increasing evidence that GLP-1 might improve endothelial and vascular function at least in part through nitric oxide dependent pathways [10,15,17,33,34]. In contrary, treatment with vildagliptin was found without beneficial effect on cardiac function in long term post-MI remodeling in rodents [35].

In our study, treatment with vildagliptin in T2DM resulted in a significant improvement in RBF and a significant decrease in the retinal arteriolar WLR. These effects on retinal microcirculation became apparent in between 12 and 24 weeks of treatment with vildagliptin, indicating some time consuming structural changes in the microvascular system. It seems conceivable, that treatment with vildagliptin interferes with vascular remodeling of small arteries in patients with T2DM. In rodents, GLP-1 receptor activation has been associated with reductions in intima hyperplasia and PDGF-induced vascular smooth muscle cell proliferation [36,37]. The clinical significance of the observed improvements in retinal microvascular blood flow and vascular architecture with regard to the development of diabetic retinopathy or other organ damage in T2DM remains unclear and needs to be clarified in pursuing studies. Nevertheless, our results are in accordance with numerous other investigations indicating an improvement in the vascular risk profile during treatment with GLP-1 receptor agonists or DPP-IV inhibitors [15,38-41]. Despite comparable glycaemic control, these effects were much weaker during treatment with glimepiride, suggesting effects of vildagliptin on retinal microvascular blood flow which go beyond glucose control.

Another important parameter in microvascular blood flow is blood viscosity, mainly driven by the elastic properties of red blood cells. The measurement of erythrocyte deformability reflects the ability of the cells to deform while entering small nutritive capillaries where the inner vessel lumen becomes less than the outer diameter of the blood cells, and the erythrocyte needs to assimilate while passing the nutritive capillary network [42,43]. Impaired erythrocyte deformability in T2DM is reported in numerous studies using different technologies [44-47], leading to the suggestion that impaired erythrocyte deformability, at least partially, accounts for the impaired tissue nutrition observed in these patients. In a recent investigation, it was shown that patients with T2DM and coronary artery disease present with impaired erythrocyte elasticity compared to those patients with coronary artery disease without T2DM [48]. An increase in blood glucose stimulates the glycosylation of the skeletal proteins beta-spectrin, ankyrin, and protein 4.2, while at the same time spectrin is damaged by oxidation [49]. Erythrocytes in patients with T2DM further show a decrease of the Na^+^K^+^-ATPase and the Ca^2+^-ATPase activity [50,51]. These alterations affect the membrane bilayer of erythrocytes in terms of their fluidity and might account for the increased rigidity of the cells. The role of elevated glucose levels on erythrocyte deformability has been described controversially [45,48,52]. The reasons for these discrepancies are matter of debate and might be explained by different patient populations or study designs. In our study, twenty-four weeks of treatment with glimepiride and vildagliptin improved erythrocyte deformability in patients with T2DM. An inverse correlation could be observed between erythrocyte deformability and fasting glucose levels (r = −0.417; p < 0.0001) as well as HbA1c (r = −0.524; p < 0.0001). Therefore, lowering of blood glucose levels in T2DM seems to improve the elastic properties of the erythrocytes independent from the kind of treatment.

In accordance with a previous study, we found an inverse correlation between plasma adiponectin levels and erythrocyte deformability (r = 0.31; p < 0.001) [52]. In hypertensive patients an inverse relationship could be observed between adiponectin levels and erythrocyte membrane fluidity as measured by an electron paramagnetic resonance and spin labeling method [53]. In addition, this study demonstrated decreased membrane fluidity in association with reduced plasma nitric oxide metabolites. Because the deformability of erythrocytes is highly dependent on their membrane fluidity [54,55], the reduction in membrane fluidity associated with low adiponectin levels might contribute to the alterations in blood rheology and tissue perfusion in patients with T2DM.

In conclusion, our study suggests that treatment with vildagliptin exert beneficial effects on retinal microvascular blood flow and retinal microvascular architecture, which could not be explained merely by improved metabolic control. In contrast, the augmentation in erythrocyte elasticity seems to correlate with improved glycaemic control and are found independent from the kind of pharmacological intervention. Treatment with vildagliptin in patients with T2DM seem to provide beneficial effects on microvascular blood flow which could be explained by glucose dependent and independent mechanisms.

### Limitations of the study

Our study was designed as an exploratory study without a priory sample size calculation. All results have to be interpreted with equal magnitude in a non-confirmatory sense. Further pursuing studies have to confirm our results and to evaluate their clinical significance for the development of vascular complications in T2DM.

## Abbreviations

AD: Arterial diameter; DPP-IV: Dipeptidyl-peptidase IV; ELISA: Enzyme linked immunosorbent assay; HPLC: High pressure liquid chromatography; LD: Lumen diameter; RBF: Retinal blood flow; T2DM: Type 2 diabetes mellitus; WLR: Wall to lumen ratio.

## Competing interests

Thomas Forst and Andreas Pfützner received research support and speaker fees from Novartis. Markus Dworak is an employee of Novartis. Christine Bernd- Zipfel, Georg Michelson, Michael Mitry, and Andrea Löffler have no competing interests. The study was supported by an unrestricted grant from Novartis.

## Authors’ contributions

CB-Z was involved in study conduct and the preparation of the manuscript. GM was involved in protocol development, assessment of retinal investigations, and the preparation of the manuscript. MD was involved in protocol development and manuscript preparation. MM and AP were involved in study conduct and manuscript preparation. AL performed statistical analysis of the results. TF was involved in protocol development, study conduct, statistical analysis, and preparation of the manuscript. All authors read and approved the manuscript.

## References

[B1] Forst T Weber MM Mitry M Schondorf T Forst S Tanis M Pfutzner A Michelson G Pilot study for the evaluation of morphological and functional changes in retinal blood flow in patients with insulin resistance and/or type 2 diabetes mellitus J Diabetes Sci Technol 2012 6 163 168 22401335 10.1177/193229681200600120 PMC3320834

[B2] Nguyen TT Kawasaki R Wang JJ Kreis AJ Shaw J Vilser W Wong TY Flicker light-induced retinal vasodilation in diabetes and diabetic retinopathy Diabetes Care 2009 32 2075 2080 10.2337/dc09-0075 19641162 PMC2768208

[B3] Cuypers MH Kasanardjo JS Polak BC Retinal blood flow changes in diabetic retinopathy measured with the Heidelberg scanning laser Doppler flowmeter Graefes Arch Clin Exp Ophthalmol 2000 238 935 941 10.1007/s004170000207 11196354

[B4] Ritt M Harazny JM Ott C Schneider MP Schlaich MP Michelson G Schmieder RE Wall-to-lumen ratio of retinal arterioles is related with urinary albumin excretion and altered vascular reactivity to infusion of the nitric oxide synthase inhibitor N-monomethyl-L-arginine J Hypertens 2009 27 2201 2208 10.1097/HJH.0b013e32833013fd 19625969

[B5] Baumann M Schwarz S Kotliar K von Eynatten M Trucksaess AS Burkhardt K Lutz J Heemann U Lanzl I Non-diabetic chronic kidney disease influences retinal microvasculature Kidney Blood Press Res 2009 32 428 433 10.1159/000264650 19996611

[B6] Awua-Larbi S Wong TY Cotch MF Durazo-Arvizu R Jacobs DR Jr Klein BE Klein R Lima J Liu K Kramer H Retinal arteriolar caliber and urine albumin excretion: the multi-ethnic study of atherosclerosis Nephrol Dial Transplant 2011 26 3523 3528 10.1093/ndt/gfr095 21398363 PMC3247797

[B7] Baleanu D Ritt M Harazny J Heckmann J Schmieder RE Michelson G Wall-to-lumen ratio of retinal arterioles and arteriole-to-venule ratio of retinal vessels in patients with cerebrovascular damage Invest Ophthalmol Vis Sci 2009 50 4351 4359 10.1167/iovs.08-3266 19339746

[B8] Harazny JM Ritt M Baleanu D Ott C Heckmann J Schlaich MP Michelson G Schmieder RE Increased wall:lumen ratio of retinal arterioles in male patients with a history of a cerebrovascular event Hypertension 2007 50 623 629 10.1161/HYPERTENSIONAHA.107.090779 17698722

[B9] Liu WJ Xie SH Liu YN Kim W Jin HY Park SK Shao YM Park TS Dipeptidyl peptidase IV inhibitor attenuates kidney injury in streptozotocin-induced diabetic rats J Pharmacol Exp Ther 2012 340 248 255 10.1124/jpet.111.186866 22025647

[B10] Ogawa S Ishiki M Nako K Okamura M Senda M Mori T Ito S Sitagliptin, a dipeptidyl peptidase-4 inhibitor, decreases systolic blood pressure in Japanese hypertensive patients with type 2 diabetes Tohoku J Exp Med 2011 223 133 135 10.1620/tjem.223.133 21304217

[B11] Crajoinas RO Oricchio FT Pessoa TD Pacheco BP Lessa LM Malnic G Girardi AC Mechanisms mediating the diuretic and natriuretic actions of the incretin hormone glucagon-like peptide-1 Am J Physiol Renal Physiol 2011 301 F355 F363 10.1152/ajprenal.00729.2010 21593184

[B12] Forst T Weber MM Pfutzner A Cardiovascular benefits of GLP-1-BasedTherapies in patients with diabetes mellitus type 2: effects on endothelial and vascular dysfunction beyond glycemic control Exp Diabetes Res 2012 2012 635472 22577369 10.1155/2012/635472 PMC3345223

[B13] Forst T Michelson G Ratter F Weber MM Anders S Mitry M Wilhelm B Pfutzner A Addition of liraglutide in patients with Type 2 diabetes well controlled on metformin monotherapy improves several markers of vascular function Diabet Med 2012 29 1115 1118 22288732 10.1111/j.1464-5491.2012.03589.x

[B14] Betteridge DJ Verges B Long-term effects on lipids and lipoproteins of pioglitazone versus gliclazide addition to metformin and pioglitazone versus metformin addition to sulphonylurea in the treatment of type 2 diabetes Int J Obes Relat Metab Disord 2005 48 2477 2481 10.1007/s00125-005-0034-1 16283239

[B15] van Poppel PC Netea MG Smits P Tack CJ Vildagliptin improves endothelium-dependent vasodilatation in type 2 diabetes Diabetes Care 2011 34 2072 2077 10.2337/dc10-2421 21788633 PMC3161271

[B16] Ussher JR Drucker DJ Cardiovascular biology of the incretin system Endocr Rev 2012 33 187 215 10.1210/er.2011-1052 22323472 PMC3528785

[B17] Shah Z Pineda C Kampfrath T Maiseyeu A Ying Z Racoma I Deiuliis J Xu X Sun Q Moffatt-Bruce S Villamena F Rajagopalan S Acute DPP-4 inhibition modulates vascular tone through GLP-1 independent pathways Vascul Pharmacol 2011 55 2 9 10.1016/j.vph.2011.03.001 21397040 PMC4845951

[B18] Kunt T Schneider S Pfutzner A Goitom K Engelbach M Schauf B Beyer J Forst T The effect of human proinsulin C-peptide on erythrocyte deformability in patients with type 1 diabetes mellitus Diabetologia 1999 42 465 471 10.1007/s001250051180 10230651

[B19] Michelson G Welzenbach J Pal I Harazny J Functional imaging of the retinal microvasculature by scanning laser Doppler flowmetry Int Ophthalmol 2001 23 327 335 10.1023/A:1014402730503 11944858

[B20] Michelson G Welzenbach J Pal I Harazny J Automatic full field analysis of perfusion images gained by scanning laser Doppler flowmetry Br J Ophthalmol 1998 82 1294 1300 10.1136/bjo.82.11.1294 9924336 PMC1722437

[B21] Izzard AS Rizzoni D Agabiti-Rosei E Heagerty AM Small artery structure and hypertension: adaptive changes and target organ damage J Hypertens 2005 23 247 250 10.1097/00004872-200502000-00002 15662208

[B22] Nguyen TT Wong TY Retinal vascular manifestations of metabolic disorders Trends Endocrinol Metab 2006 17 262 268 10.1016/j.tem.2006.07.006 16890449

[B23] Sabanayagam C Shankar A Koh D Chia KS Saw SM Lim SC Tai ES Wong TY Retinal microvascular caliber and chronic kidney disease in an Asian population Am J Epidemiol 2009 169 625 632 19092170 10.1093/aje/kwn367

[B24] Porta M Grosso A Veglio F Hypertensive retinopathy: there’s more than meets the eye J Hypertens 2005 23 683 696 10.1097/01.hjh.0000163131.77267.11 15775767

[B25] Nagaoka T Yoshida A Relationship between retinal blood flow and renal function in patients with type 2 diabetes and chronic kidney disease Diabetes Care 2012 36 957 961 23204249 10.2337/dc12-0864 PMC3609484

[B26] Schmieder RE Ritt M Wall-to-lumen ratio of retinal arterioles: a reproducible, valid and noninvasive approach for evaluation of early arteriolar changes in arterial hypertension in vivo J Hypertens 2012 30 1108 1110 10.1097/HJH.0b013e328353f85a 22573078

[B27] Ritt M Harazny JM Ott C Raff U Schneider MP Michelson G Schmieder RE Basal nitric oxide activity is an independent determinant of arteriolar structure in the human retinal circulation J Hypertens 2011 29 123 129 10.1097/HJH.0b013e3283406940 21045732

[B28] Sakamoto M Nishimura R Irako T Tsujino D Ando K Utsunomiya K Comparison of vildagliptin twice daily vs. sitagliptin once daily using continuous glucose monitoring (CGM): crossover pilot study (J-VICTORIA study) Cardiovasc Diabetol 2012 11 92 10.1186/1475-2840-11-92 22867630 PMC3471040

[B29] Bluher M Kurz I Dannenmaier S Dworak M Efficacy and safety of vildagliptin in clinical practice-results of the PROVIL-study World J Diabetes 2012 3 161 169 23125906 10.4239/wjd.v3.i9.161 PMC3487174

[B30] Forst T Dworak M Berndt-Zipfel C Loffler A Klamp I Mitry M Pfutzner A Effect of vildagliptin compared to glimepiride on postprandial proinsulin processing in the beta cell of patients with type 2 diabetes mellitus Diabetes Obes Metab 2013 [Epub ahead of print] 10.1111/dom.12063 23384119

[B31] Jose T Inzucchi SE Cardiovascular effects of the DPP-4 inhibitors Diab Vasc Dis Res 2012 9 109 116 10.1177/1479164111436236 22337893

[B32] Heo KS Fujiwara K Abe J Glucagon-like peptide-1 and its cardiovascular effects Curr Atheroscler Rep 2012 14 422 428 10.1007/s11883-012-0265-9 22878937

[B33] Ban K Noyan-Ashraf MH Hoefer J Bolz SS Drucker DJ Husain M Cardioprotective and vasodilatory actions of glucagon-like peptide 1 receptor are mediated through both glucagon-like peptide 1 receptor-dependent and -independent pathways Circulation 2008 117 2340 2350 10.1161/CIRCULATIONAHA.107.739938 18427132

[B34] Golpon HA Puechner A Welte T Wichert PV Feddersen CO Vasorelaxant effect of glucagon-like peptide-(7–36)amide and amylin on the pulmonary circulation of the rat Regul Pept 2001 102 81 86 10.1016/S0167-0115(01)00300-7 11730979

[B35] Yin M Sillje HH Meissner M van Gilst WH de Boer RA Early and late effects of the DPP-4 inhibitor vildagliptin in a rat model of post-myocardial infarction heart failure Cardiovasc Diabetol 2011 10 85 10.1186/1475-2840-10-85 21955567 PMC3198901

[B36] Goto H Nomiyama T Mita T Yasunari E Azuma K Komiya K Arakawa M Jin WL Kanazawa A Kawamori R Fujitani Y Hirose T Watada H Exendin-4, a glucagon-like peptide-1 receptor agonist, reduces intimal thickening after vascular injury Biochem Biophys Res Commun 2011 405 79 84 10.1016/j.bbrc.2010.12.131 21215253

[B37] Murthy SN Hilaire RC Casey DB Badejo AM McGee J McNamara DB Kadowitz PJ Fonseca VA The synthetic GLP-I receptor agonist, exenatide, reduces intimal hyperplasia in insulin resistant rats Diab Vasc Dis Res 2010 7 138 144 10.1177/1479164109360269 20382777

[B38] Derosa G Ragonesi PD Carbone A Fogari E D’Angelo A Cicero AF Maffioli P Vildagliptin action on some adipocytokine levels in type 2 diabetic patients: a 12-month, placebo-controlled study Expert Opin Pharmacother 2012 13 2581 2591 10.1517/14656566.2012.734499 23121473

[B39] Gallwitz B Rosenstock J Rauch T Bhattacharya S Patel S von Eynatten M Dugi KA Woerle HJ 2-year efficacy and safety of linagliptin compared with glimepiride in patients with type 2 diabetes inadequately controlled on metformin: a randomised, double-blind, non-inferiority trial Lancet 2012 380 475 483 10.1016/S0140-6736(12)60691-6 22748821

[B40] Cobble ME Frederich R Saxagliptin for the treatment of type 2 diabetes mellitus: assessing cardiovascular data Cardiovasc Diabetol 2012 11 6 10.1186/1475-2840-11-6 22248301 PMC3277488

[B41] Monami M Dicembrini I Martelli D Mannucci E Safety of dipeptidyl peptidase-4 inhibitors: a meta-analysis of randomized clinical trials Curr Med Res Opin 2011 27 Suppl 3 57 64 22106978 10.1185/03007995.2011.602964

[B42] Mohandas N Chasis JA Shohet SB The influence of membrane skeleton on red cell deformability, membrane material properties, and shape Semin Hematol 1983 20 225 242 6353591

[B43] Yedgar S Koshkaryev A Barshtein G The red blood cell in vascular occlusion Pathophysiol Haemost Thromb 2002 32 263 268 10.1159/000073578 13679654

[B44] Brown CD Ghali HS Zhao Z Thomas LL Friedman EA Association of reduced red blood cell deformability and diabetic nephropathy Kidney Int 2005 67 295 300 10.1111/j.1523-1755.2005.00082.x 15610255

[B45] Shin S Ku YH Ho JX Kim YK Suh JS Singh M Progressive impairment of erythrocyte deformability as indicator of microangiopathy in type 2 diabetes mellitus Clin Hemorheol Microcirc 2007 36 253 261 17361027

[B46] Babu N Influence of hypercholesterolemia on deformability and shape parameters of erythrocytes in hyperglycemic subjects Clin Hemorheol Microcirc 2009 41 169 177 19276514 10.3233/CH-2009-1165

[B47] Tsukada K Sekizuka E Oshio C Minamitani H Direct measurement of erythrocyte deformability in diabetes mellitus with a transparent microchannel capillary model and high-speed video camera system Microvasc Res 2001 61 231 239 10.1006/mvre.2001.2307 11336534

[B48] Keymel S Heiss C Kleinbongard P Kelm M Lauer T Impaired red blood cell deformability in patients with coronary artery disease and diabetes mellitus Horm Metab Res 2011 43 760 765 22009370 10.1055/s-0031-1286325

[B49] Le DC Khodabandehlou T Vimeux M Relationship between hemorheological and microcirculatory abnormalities in diabetes mellitus Diabete Metab 1994 20 401 404 7843471

[B50] Zimny S Dessel F Ehren M Pfohl M Schatz H Early detection of microcirculatory impairment in diabetic patients with foot at risk Diabetes Care 2001 24 1810 1814 10.2337/diacare.24.10.1810 11574447

[B51] Cicco G Pirrelli A Red blood cell (RBC) deformability, RBC aggregability and tissue oxygenation in hypertension Clin Hemorheol Microcirc 1999 21 169 177 10711739

[B52] Forst T Weber MM Lobig M Lehmann U Muller J Hohberg C Friedrich C Fuchs W Pfutzner A Pioglitazone in addition to metformin improves erythrocyte deformability in patients with Type 2 diabetes mellitus Clin Sci (Lond) 2010 119 345 351 10.1042/CS20100161 20509857

[B53] Tsuda K Adiponectin and membrane fluidity of erythrocytes in normotensive and hypertensive men Obesity (Silver Spring) 2006 14 1505 1510 10.1038/oby.2006.173 17030960

[B54] Tsuda K Nishio I Membrane fluidity and hypertension Am J Hypertens 2003 16 259 261 10.1016/S0895-7061(02)03257-0 12620708

[B55] Zicha J Kunes J Devynck MA Abnormalities of membrane function and lipid metabolism in hypertension: a review Am J Hypertens 1999 12 315 331 10.1016/S0895-7061(98)00178-2 10192236

